# Unilateral phrenic nerve lesion in Lyme neuroborreliosis

**DOI:** 10.1186/1471-2466-13-4

**Published:** 2013-01-18

**Authors:** Marija Djukic, Jörg Larsen, Paul Lingor, Roland Nau

**Affiliations:** 1 Department of Neuropathology, University Medical Centre Goettingen, Robert-Koch-Str. 40, 37075, Goettingen, Germany; 2 Department of Clinical Radiology, Weende Teaching Hospitals, An der Lutter 24, 37075, Goettingen, Germany; 3 Department of Neurology, University Medical Centre Goettingen, Robert-Koch-Str. 40, 37075, Goettingen, Germany; 4 Department of Geriatrics, Weende Teaching Hospitals, An der Lutter 24, 37075, Goettingen, Germany

**Keywords:** Phrenic nerve palsy, Unilateral, Lyme neuroborreliosis, Dyspnoea, Diaphragm, Doxycycline, Ceftriaxone

## Abstract

**Background:**

Among a variety of more common differential diagnoses, the aetiology of acute respiratory failure includes Lyme neuroborreliosis.

**Case presentation:**

We report an 87-years old huntsman with unilateral phrenic nerve palsy as a consequence of Lyme neuroborreliosis.

**Conclusion:**

Although Lyme neuroborreliosis is a rare cause of diaphragmatic weakness, it should be considered in the differential workup because of its potentially treatable nature.

## Background

Lyme neuroborreliosis (LNB) is the neurological manifestation of the systemic infection caused by the spirochete *Borrelia burgdorferi (BB)* sensu lato. Clinical features of LNB are diverse and differ among European and American patients, most probably because of differences in the distribution of *Borrelia* species in Europe and North America [1,2]. The most common neurological manifestation of LNB in European adults is polyradiculoneuritis (Bannwarth’s syndrome) [3], which typically occurs in patients older than 50 years, while in younger patients a meningitic syndrome of LNB is common [4]. Although several studies indicate that severe paralysis may occur in LNB [1], diaphragmatic weakness as a symptom of LNB is rare [2]. Here we report a case of LNB leading to a reduction of vital capacity and severe dyspnoea as a consequence of unilateral paralysis of the diaphragm.

## Case presentation

An 87-years old huntsman presented with severe headache, shooting left-sided thoracic pain, fatigue and vertigo. Neurological examination revealed partial dysfunction of the right abducens nerve. During the course of the disease a right facial palsy, with dysarthria and dysphagia as a consequence of facial weakness developed. Although the patient reported multiple previous tick bites, he had never noticed an erythema migrans. Brain magnetic resonance imaging (MRI) without and with contrast medium was unremarkable. The cerebrospinal fluid (CSF) analysis revealed a lymphocytic pleocytosis (129 cells/μl), an elevated lactate level of 2,6 mmol/l, a protein level of 1324 mg/l and an intrathecal *Borrelia burgdorferi* (*BB*)-IgG antibodies synthesis (*BB*-specific antibody index for IgG 5.0 and for IgM 0.8). *BB*-PCR of CSF was negative. The negative CSF PCR for *BB*-specific DNA does not exclude the diagnosis, as the sensitivity of PCR in LNB is very low [5].

**Figure 1:**
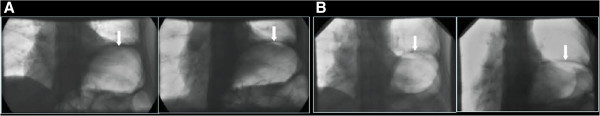
**Frontal projection screening fluoroscopy images of the patient’s diaphragms during spontaneous ventilation in Lyme neuroborreliosis.** At the time when the patient’s dyspnoea developed (Panel **A**), there was no significant change in the position and shape of the left hemidiaphragm between expiration (left) and inspiration (right; arrows). Six months following treatment (Panel **B**), there was relative depression and, specifically, flattening of the left hemidiaphragm on inspiration (right as compared to left image; arrows). Note relative splinting of the left hemidiaphragm due to gaseous distension of the gastric fundus (all images).

These findings led to the diagnosis of acute LNB. The patient was treated with intravenous ceftriaxone at a dose of 2 g/d for two weeks. His symptoms of headache, dysarthria and dysphagia improved, facial and abducens nerve palsies recovered slowly. Two days following his discharge from hospital, the patient developed dyspnoea and a mild cough. The clinical diagnosis upon out-patient review was pneumonia, and the patient was admitted again. Biplanar chest radiography showed a partial atelectasis of the left lower lobe, and screening fluoroscopy showed paralysis of the left hemidiaphragm (Figure 1A), but no air-space shadowing indicative of pneumonia. No leucocytosis or elevated C-reactive protein were found on laboratory analysis. Oral antibiotic treatment with doxycyclin (200 mg/d) was commenced and continued for 14 days, since LNB was considered the probable cause of the diaphragmatic weakness. During the following two weeks dyspnoea did, however, not resolve. Pulmonary function tests revealed a vital capacity of 2.6 l. To exclude a mass lesion of the lung, chest computerised tomography (CT) was done. It confirmed elevation of the left hemidiaphragm and partial atelectasis of the left lower lobe, associated with a small pleural effusion without detection of other lung lesions. Bronchoscopy was unremarkable, so that no other cause for the paralysis was found. Diaphragmatic paralysis in our patient became first evident after the presentation with typical clinical neurological features of acute LNB and CSF findings indicating a recent infection with *BB*.

Six months later, the patient had no dyspnoea at rest but continued to be short of breath on exertion. Repeat fluoroscopic examination showed partial recovery of the diaphragmatic weakness (Figure 1B). The abducens nerve palsy had recovered completely, but a mild facial palsy remained.

Lyme borreliosis may involve all parts of the nervous system [1] with as many as 15–20% of infected patients developing neurological symptoms [6]. These usually occur in the first three months following the infection. Diaphragmatic weakness has been associated with trauma, malignant compression or infiltration, metabolic, inflammatory and other disorders, but rarely with Lyme disease [7]. Early recognition and appropriate treatment of diaphragmatic paralysis caused by *BB* is important in order to shorten the duration of severe respiratory symptoms [7].

Unilateral diaphragmatic paralysis is a respiratory disorder, and many neurological diseases, such as spinal cord injury, motor neuron disease, and carcinomatous infiltration of the phrenic nerve are included in its differential diagnosis [8]. The development of respiratory compromise due to peripheral nerve involvement in patients with LNB is rare. To our knowledge, only 8 such patients have been described in the literature [7,9-13]. The severity of the respiratory involvement varies from mild symptoms to the need for artificial ventilation for several months [7,13]. In the last published report, as many as three patients with diaphragmatic weakness caused by LNB presented over a one-year period. This suggests that the incidence of LNB as a cause of diaphragmatic weakness is underestimated [13].

Diaphragmatic weakness in LNB results from radiculopathy of the nerve roots C3-C5 or neuropathy of the phrenic nerve [13]. The pathogenesis of the nervous system involvement is uncertain: both direct infection with stimulation of Toll-like receptors [14] as well as immunological mechanisms with stimulation of T- and B-cells [15] may play a role.

As in the present case, the clinical diagnosis of LNB is based on a combination of history, typical clinical symptoms, the detection of *BB* antibodies in serum and CSF and calculation of the *BB*-specific antibody index [4]. *BB* antibody tests in serum may be negative in early infection. Conversely, serum antibodies against *BB* are detectable in up to 25% of healthy persons in Europe [16]. In some patients, antibodies against *BB* may be detectable in CSF slightly earlier than in serum [4]. Both *BB*-specific antibodies in serum and *BB*-specific intrathecal antibody synthesis can persist for years after eradication of the infection [17]. In recent years, the B lymphocyte chemoattractant chemokine CXCL13 has been identified as a potentially important biomarker for the diagnosis of acute LNB [18,19]. CXCL13, however, seems to be the major determinant for B cell recruitment to the CNS compartments in different neuroinflammatory diseases and not just in LNB [20]. CXCL13 levels in the CSF rather reflect a strong humoral immune response in the CNS compartments than being specific for a particular disease entity [20].

The marked inflammatory response in CSF (lymphopleocytosis and production of *BB*-specific antibodies and the absence of other causes) indicate an acute Lyme neuroborreliosis as the reason for respiratory failure in the present case. Irrespective of appropriate antibiotic therapy, diaphragmatic dysfunction in our patient persisted for a long period. Treatment with adequate antimicrobial regimens is usually effective in LNB [21] but some symptoms may persist longer despite antibiotic treatment, and the recovery can be incomplete [22-24].

## Conclusion

LNB should be considered in the differential diagnosis of acute respiratory failure due to diaphragmatic paralysis since it requires adequate antibiotic treatment. It is important in clinical practice to consider Lyme neuroborreliosis also in patients presenting with respiratory failure as an isolated syndrome.

### Consent

Written informed consent was obtained from the patient for publication of this Case report and any accompanying images. A copy of the written consent is available for review by the Series Editor of this journal.

## Competing interests

There are non-financial competing interests.

## Authors’ contribution

MD and RN made substantial contributions to conception and design, analysis and interpretation of data. JL and PL were involved in drafting the manuscript, revising it critically for the intellectual content. All authors read and approved the final manuscript.

## Pre-publication history

The pre-publication history for this paper can be accessed here:

http://www.biomedcentral.com/1471-2466/13/4/prepub
